# The role of hormonal and reproductive events on cognitive aging in a cohort of female civil servants: The Whitehall II Study

**DOI:** 10.1002/alz70860_101675

**Published:** 2025-12-23

**Authors:** Sima T Toopchiani, Miia Kivipelto, Lefkos T Middleton, Oliver J Robinson, Shireen Sindi

**Affiliations:** ^1^ The Ageing Epidemiology (AGE) Research Unit, School of Public Health, Imperial College London, London, United Kingdom, London, United Kingdom; ^2^ Karolinska Institutet, Department of Neurobiology, Care Sciences and Society, Division of Clinical Geriatrics, Center for Alzheimer Research, Stockholm, Sweden; ^3^ The Ageing Epidemiology (AGE) Research Unit, School of Public Health, Imperial College London, London, England, United Kingdom

## Abstract

**Background:**

Sex differences in dementia prevalence and cognitive decline have been widely reported, yet the role of sex‐specific risk factors is still unclear. We aimed to investigate the relationship between hormonal/reproductive events and cognitive performance in women.

**Methods:**

We analyzed data from 1,398 female civil servants (aged 45‐68 years) participating in the Whitehall II study. We assessed the longitudinal associations between baseline hormonal and reproductive events on cognitive performance including memory (immediate recall) and fluency (phonemic fluency test) over a 25‐year follow‐up (1997‐2015). Mixed models including an interaction term to model change with time, were used to analyse repeated cognitive scores (standardized to baseline scores), adjusted for demographic (age, ethnicity), socioeconomic (education, income, occupation, marital status), vascular (body mass index, cardiovascular disease, hypertension, diabetes, stroke), lifestyle (smoking, alcohol, sleep, diet) and other factors (depression, anxiety, cancer).

**Results:**

Steeper declines in phonemic fluency were observed in women who had been pregnant (adjusted mean difference: ‐0.05, 95% confidence interval (95% CI)‐0.05,‐0.04) (reference: had not been pregnant), had two (‐0.03, 95% CI ‐0.03,‐0.02) or more children (‐0.05, 95% CI ‐0.05,‐0.05) (reference: no children), had their first child at 30‐34 years (‐0.06, 95%CI ‐0.07, ‐0.04) (reference: aged 20‐29 years), experienced cycle lengths of ≤23 days (‐0.02, 95%CI ‐0.03,‐0.009) (reference: cycle length 23‐35 days), underwent menopause (‐0.1, 95%CI ‐0.1,‐0.1) (reference: still menstruating), or stopped menstruating at 40‐49 years (‐0.06, 95% CI ‐0.07, ‐0.06) (reference: stopped menstruating aged ≥50 years). In contrast, having children at age 35+ years (0.05, 95% CI 0.03,0.06), oral contraceptive use (0.07, 95% CI 0.06,0.08), and cycle lengths of 35+ days (0.1, 95% CI 0.1,0.1) were associated with less declines over 25 years. Similar steeper memory declines were observed among women who had been pregnant (‐0.06, 95% CI ‐0.06,‐0.05), had 3+ children (‐0.1, 95% CI ‐0.2,‐0.1), had a first pregnancy at 30‐34 years (‐0.05, 95% CI ‐0.06,‐0.04) and underwent menopause (‐0.07, 95% CI ‐0.07,‐0.06).

**Conclusion:**

These findings demonstrate potential long‐term associations between hormonal and reproductive events on cognitive aging. Further research is needed on the underlying mechanisms and the potential interactions with risk factors that exacerbate these associations.

